# Presence of Fatty Liver and the Relationship between Alcohol Consumption and Markers of Inflammation

**DOI:** 10.1155/2015/278785

**Published:** 2015-02-18

**Authors:** Martin Kächele, Stefan Wolff, Wolfgang Kratzer, Mark Haenle, Jörg Homann, Gerlinde Trischler, Wolfgang Koenig, Armin Imhof

**Affiliations:** ^1^Department of Internal Medicine II (Cardiology, Angiology, Pulmonology, Sports, and Rehabilitation Medicine), University of Ulm Medical Centre, Albert-Einstein-Allee 23, 89081 Ulm, Germany; ^2^Department of Internal Medicine I (Gastroenterology, Endocrinology, and Nephrology), University of Ulm Medical Centre, Albert-Einstein-Allee 23, 89081 Ulm, Germany

## Abstract

*Background and Aims*. Local and systemic inflammation represent a major feature of atherosclerotic cardiovascular disease (CVD) and are also linked to nonalcoholic fatty liver disease (NAFLD). Studies indicate that NAFLD might be a risk factor for CVD whereas low-to-moderate alcohol consumption is associated with lower cardiovascular morbidity and mortality compared to abstainers and heavy drinkers. We hypothesize that FLD interacts with the effect of alcohol intake on markers of inflammation, and thus potentially on cardiovascular risk. *Methods and Results*. We evaluated alcohol consumption, markers of inflammation and sonographic criteria of FLD in 515 subjects, representing a subsample of a cross-sectional population based study (Echinococcus multilocularis and Internal Diseases in Leutkirch (EMIL) Study). Presence of FLD was markedly reduced in subjects drinking 0–20 g alcohol/d (19%), compared to nondrinkers (35%) and heavy drinkers (34–44.9%). Serum concentrations of inflammatory markers were substantially higher in subjects with FLD. However, presence of FLD showed no effect on the association between alcohol consumption and inflammatory biomarkers. *Conclusions*. Based on data from a population-based sample, there is no evidence for a link between FLD, alcohol consumption, and inflammatory cardiovascular risk markers. However, larger prospective studies are needed to confirm this.

## 1. Introduction

Inflammation is associated with atherosclerosis as well as with fatty liver disease (FLD). Both diseases are common in the western world [1] and several studies indicate that there could be a link between both disorders [2].

### 1.1. Atherosclerosis

Atherosclerosis is characterized by a complex pathophysiology that is initiated by endothelial dysfunction. Inflammation causes formation of vascular atheromas and thinning of the plaque's fibrous cap; unstable plaques can rupture and cause coronary thrombosis resulting in myocardial infarction [3].

### 1.2. NAFLD

Fatty liver disease is considered to be the hepatic manifestation of metabolic diseases, summarized as the metabolic syndrome (nonalcoholic fatty liver disease). Severe forms are characterized by an inflammation of the hepatic tissue, induced by inflammatory cell invasion and cytokine production, leading to hepatocyte necrosis and fibrosis [4].

NAFLD and CVD are inflammatory diseases and a number of common cytokines are involved in the inflammatory process of both diseases [5, 6]. Low grade vascular inflammation in CVD is associated with systemically measurable markers of inflammation. These biomarkers have been shown to independently predict cardiovascular events. A variety of potentially predictive biomarkers have been identified so far [7] (see below); some of these might play an active role in the initiation and progression of CVD and might also be linked to NAFLD, such as adiponectin or interleukin- (IL-) 6 [5].

### 1.3. Alcohol and Cardiovascular Disease

Alcohol intake is associated with the risk of all-cause mortality in U- or J-shaped manner, primarily through reduction of the risk for coronary heart disease (CHD) [8, 9]. In several meta-analyses, the lowest risk has been found among those with light-to-moderate alcohol consumption (5–25 g/day) [10, 11]. Some studies could demonstrate that moderate alcohol consumption is associated with lower concentrations of circulating inflammatory biomarkers compared to nondrinkers and those with high alcohol consumption [12, 13]. Thus, it has been suggested that potential beneficial effects of moderate alcohol consumption might be mediated at least in part by lowering the inflammatory burden in different stages of atherosclerotic disease [14, 15].

### 1.4. NAFLD and Cardiovascular Risk

As moderate alcohol consumption seems to have anti-inflammatory effects, one might speculate that it may also reduce the risk of developing NAFLD.

Further, NAFLD has been shown to be associated with an increased risk for cardiovascular events [16, 17]. There is an ongoing debate whether NAFLD is an independent risk factor for cardiovascular disease or a concomitant disease [5, 18].

Given the link between these two inflammatory entities, the presence of NAFLD might affect concentrations of circulatory inflammatory biomarkers. Further, it might interact with the effect of alcohol intake on markers of inflammation and thus also on the cardiovascular risk.

### 1.5. Inflammatory Biomarkers

Lipoprotein oxidation, especially of low-density lipoprotein (oxLDL), is a crucial step in the development of early atherosclerotic lesions. In cohort studies, oxLDL was a strong predictor for coronary heart disease [19].

Adiponectin mediates insulin-sensitivity and has anti-inflammatory and antiatherogenic effects. Low serum levels of adiponectin can be found in patients with type 2 diabetes or coronary artery disease. C-reactive protein (CRP), an acute phase-protein, is produced by the liver. Increased serum levels in noninfectious situations, as measured by high sensitivity assays, are predictive for the risk of cardiovascular events [20].

Interleukin-6 (IL-6) potently promotes the secretion of other proinflammatory cytokines. IL-6 baseline levels are independently associated with future cardiovascular endpoints in apparently healthy subjects [21].

Fibrinogen also shows an association between elevated baseline levels and an increased cardiovascular risk. Fibrinogen levels have been shown to be inversely correlated with alcohol consumption [12, 13].

E-selectin belongs to the family of adhesion molecules. Elevated serum levels are thought to reflect early stages of atherosclerotic vascular disease [7, 22].

### 1.6. Aim of the Study

We examined the association between alcohol consumption and prevalence of NAFLD, their interaction with serum levels of different biomarkers predictive for cardiovascular disease, or its complications in a large cross-sectional population-based study.

## 2. Methods

### 2.1. Study Population and Data Collection

A subsample of 515 men and women aged 18–49 was randomly selected according to the amount of self-reported alcohol consumption from a population-based cross-sectional survey including 2445 individuals in the area of Leutkirch in Southern Germany. This survey was primarily designed to estimate the prevalence of* Echinococcus multilocularis* and Internal Diseases in Leutkirch (EMIL) Study. The overall participation rate was 62.8%. A standardized interview was performed by trained personnel, including sociodemographic data, medical history, and lifestyle habits, including physical activity, drug history, and alcohol consumption. To estimate alcohol consumption, each subject was interviewed about the amount of beer, wine, and spirits he or she had consumed on the previous workday and over the last weekend. Total alcohol intake was calculated by multiplying weekday consumption by five and adding this figure to weekend consumption. An average amount of alcohol intake in grams per day was calculated. This recall method had been validated in a subsample of 899 male participants of the first MONICA Augsburg survey in 1984/85 [23]. Ex-drinkers were identified and separately characterized but excluded from the analyses. Body height (in m), body weight (in kg), waist circumference, and hip circumference were measured in a standardized manner. Body mass index (kg/m²), waist-to-hip ratio, and daily alcohol intake were calculated. Physical inactivity was defined as being physically active during leisure time neither in summer nor in winter. Collection of data on physical activity was based on standardized and validated questions used in previous large epidemiological studies. This has been described in detail elsewhere [24].

All subjects gave informed, written consent, and all protocols and collection procedures were approved by the relevant institutional committees.

### 2.2. Abdominal Ultrasound

All participants underwent a real-time ultrasonographic examination of the liver for detection of typical signs of fatty liver disease and other pathologies performed by a single investigator (HDI 5000, Advanced Technology Laboratories, Philips Medical Systems, Bothell, WA, USA). The diagnosis of a fatty liver was based on the finding of a bright liver tissue signal with fine tightly packed echoes, making it significantly more echogenic compared to the adjacent right kidney [25, 26]. Liver size was measured from cranial to caudal edge in the midclavicular line.

### 2.3. Laboratory Methods

Fasting blood was collected from the antecubital vein in the supine position with minimal suction and short-term occlusion. Plasma was obtained by centrifugation at 3,000 g for 15 min and was stored at −20°C within 90 min and then stored at −70°C until analysis. All laboratory analyses were done in a blinded fashion. Liver enzymes were measured the same day photometrically from Lithium-heparin plasma on a Dimension XL (Dade Behring, Marburg, Germany). HDL-cholesterol was measured enzymatically on a Dimension RxL (Dade Behring, Marburg, Germany). Further measurements were performed using a highly sensitive ELISA for IL-6 (R&D Systems, Wiesbaden, Germany; range 0–10 pg/mL; coefficient of variation (CV) 17.8%), an ELISA for E-selectin (R&D Systems, Wiesbaden, Germany; range 0–9.69 ng/mL; CV 7.92%), and also one for oxLDL (Mercodia, USA; range 0–7.6 U/L; CV 7.99%). A high-sensitivity assay (latex-enhanced nephelometry; CV 4.26% for apolipoprotein control and 5.85% for rheuma control) was used for CRP, also nephelometry for fibrinogen (Dade Behring, Marburg, Germany; CV 5.73%). Adiponectin concentrations were measured using an ELISA (Quantikine, R&D Systems, Wiesbaden, Germany; 0–250 ng/mL range; CV 8,93%).

### 2.4. Statistical Analysis

Sociodemographic, clinical, and biochemical characteristics were analyzed in a descriptive manner. Adjusted geometric mean for CRP and adjusted arithmetic mean for the other inflammatory markers were calculated in categories of daily alcohol intake from multiple linear regression analyses. Known or presumed potential confounders including age (four categories), sex, smoking status (current, ex, and never), body mass index (continuous), HDL-cholesterol (continuous), formal education, and physical activity (two categories) were forced into all models. All tests performed were two-sided, and a *P* value < 0.05 was considered statistically significant. All computations were performed using SAS software, Release 8.2 (SAS Institute Inc. Cary, NC, USA).

## 3. Results

### 3.1. Study Population Characteristics

Characteristics of the subgroups, divided according to the quantity of alcohol consumption, are shown in Table 1. Those participants, who reported former alcohol use, were excluded. This group showed a heterogeneous characteristics pattern, which differed notably from that of nondrinkers (data not shown). There was no significant difference between groups in terms of age, smoking history, hypertension, BMI, physical inactivity, intake of aspirin or statins, and presence of coronary artery disease. The level of education decreased in groups with higher alcohol consumption.

### 3.2. Alcohol and the Prevalence of FLD

Prevalence of fatty liver disease was far the lowest among participants reporting consumption of 0–20 g of alcohol per day (19.0%). Higher or no intake (abstinence) was associated with a substantially higher prevalence of NAFLD (35.0% for 0 g/d, 34.0% for 20–40 g/d, 38.6% for 40–60 g/d, and 44.9% for >60 g/d).

### 3.3. Alcohol and Liver Enzymes

Serum levels of GGT as well as of ALT showed a slight increase with more alcohol consumption (12 to 36 U/l for GGT *P* < 0.0001, 14 to 19 U/l ALT *P* = 0.0004). In the “moderate drinker” group (0–20 g/d), though, serum levels were comparable to those of nondrinkers (Table 2).

When subgroups were divided by presence or absence of fatty liver disease, serum levels for ALT were consistently higher in people with NAFLD.

HDL-C did not significantly differ between groups.

### 3.4. Fatty Liver Disease and Inflammatory Markers

Next, we analyzed pro- and anti-inflammatory biomarkers with regard to the presence or absence of FLD (Table 3). Across all subgroups, concentrations of proinflammatory biomarkers were substantially higher in subjects with NAFLD. Accordingly, concentrations of adiponectin, an anti-inflammatory marker, were lower.

### 3.5. Alcohol and the Association of Biomarkers and Fatty Liver Disease

Of all biomarkers, only IL-6 showed an approximately linear association with alcohol consumption, with a slight “notch” at 40–60 g alcohol/d. We found a clear association between alcohol intake and inflammatory markers neither in males nor among females.

Serum concentrations of adiponectin were lower in subjects with NAFLD. Low levels of adiponectin were found in nondrinkers (5.04 *μ*g/mL with FLD, 7.59 *μ*g/mL without FLD), whereas levels at low-moderate alcohol consumption were significantly higher (7.74 *μ*g/mL with FLD, 8.91 *μ*g/mL without FLD). Particularly in those with FLD, the increased serum level of adiponectin at moderate alcohol consumption was remarkable. With higher alcohol intake, the adiponectin serum levels decreased again in this group.

## 4. Discussion

### 4.1. Study Population Characteristics

The present study analyzed the associations between alcohol consumption and the prevalence of FLD and of pro- and anti-inflammatory biomarkers in a cross-sectional sample of a general urban population. Nine out of ten people consuming more than 40 g alcohol per day were men, whereas, in the nondrinkers and moderate drinkers group, men represented less than 50%.

### 4.2. Alcohol and the Prevalence of FLD

The low prevalence of NAFLD among subjects with moderate alcohol consumption represents the most intriguing finding in this study. The prevalence of FLD in the group consuming less than 20 g alcohol per day was 19%. Below 20 g per day, alcohol consumption is in general not considered to cause FLD [4]. In the total study population, mean BMI ranged from 24.8 to 26.3 kg/m². Although the data had been adjusted for potential confounders like BMI, education, physical inactivity, and age, it cannot be completely ruled out that subjects with a generally healthier lifestyle and a higher socioeconomic status are overrepresented in the moderate drinker group. The prevalence of nonalcoholic fatty liver disease found in this study is comparable to that of the Dionysos Nutrition and Liver Study with 20 to 25% [1].

A recent analysis of a large, representative cross-sectional study (NHANES III), which investigated the association between consumption of different alcoholic beverages up to 10 g daily and unexplained liver enzyme elevation (ALT), found the lowest prevalence of ALT elevation among wine drinkers [27]. Even though the authors reported differences in the prevalence of FLD depending on the type of beverage, it was almost the highest in nondrinkers compared to subjects drinking any alcohol. Interestingly, in a cross-sectional population with biopsy proven NAFLD (none and moderate drinkers), moderate alcohol consumption was associated with lower odds for steatohepatitis and fibrosis compared to nondrinkers [28]. In another study with a sample of 132 severely obese patients, who underwent liver biopsy, however, no differences in the prevalence or severity of fatty liver disease were seen in relation to the level of alcohol consumption [29]. However, among these morbid patients with a mean BMI of 43 kg/m², only about 10% showed no FLD and the majority (>80%) had severe FLD across all categories of alcohol intake. In these subjects, the underlying metabolic syndrome might have been the driving force of hepatic pathophysiology.

### 4.3. Alcohol and Liver Enzymes

GGT is a classical marker for excessive alcohol consumption. In this study, we found a consistent correlation between GGT serum concentrations and self-reported alcohol intake. Serum levels for nondrinkers and subjects with low-to-moderate alcohol consumption were not different, indicating a low likelihood for liver damage. It is possible that elevated serum levels of GGT itself contribute to the increased risk for cardiovascular events in heavy drinkers. Recently, GGT has been proposed as an independent cardiovascular risk marker and several mechanisms have been suggested to explain its active role in atherogenesis [30].

### 4.4. Fatty Liver Disease and Biomarkers of Inflammation

Serum levels of all biomarkers were substantially higher in subjects with FLD. As shown in Table 3, there is an increase in serum levels in subjects with FLD of all proinflammatory biomarkers, namely, fibrinogen, ox-LDL, IL-6, CRP, and E-selectin. Adiponectin, as expected, shows an inverse association. The presence of FLD thus constantly increases the inflammatory burden and thereby might be correlated with an increased cardiovascular risk. However, most likely FLD does not alter the known association between alcohol consumption and cardiovascular risk. CRP, for example, has been described to be associated with alcohol consumption in a J-shaped manner. Yet, we were not able to identify a clear J-shaped association in individuals neither with nor without FLD. At present, the CRP lowering effect of alcohol is still discussed controversially. A meta-analysis of interventional studies by Brien et al. including 5 studies with a total of only 186 participants was not able to find a significant reduction of CRP in moderate drinkers [13].

Also, association curves for all other biomarkers did not reflect the known J-shaped association. Therefore, in this study, the effect of alcohol on inflammatory biomarkers is not conclusive, which might be due, at least in part, to the small sample size in each category.

A large number of cardiovascular biomarkers have been suggested to be lowered in subjects with moderate alcohol intake. Fibrinogen and CRP are elevated at all stages of the atherosclerotic process. All other biomarkers chosen in this study (IL-6, E-selectin, and oxLDL) reflect different stages of vascular inflammation. Yet, it is not fully understood how moderate amounts of alcohol reduce cardiovascular mortality, although anti-inflammatory mechanisms might play a role. A meta-analysis of prospective studies on alcohol intake and its effects on biomarkers by Brien et al. showed that high levels of HDL-cholesterol and adiponectin and low levels of fibrinogen correlated best with moderate alcohol intake [13]. These markers might be the key to understand antiatherogenic mechanisms and the role of alcohol in other inflammatory conditions, such as fatty liver disease. Finally, the most important limitation of our study is its cross-sectional design not allowing causal inferences. Larger prospective studies are needed to shed light on this complex area.

## 5. Conclusion

The present study demonstrates that low-to-moderate alcohol consumption is associated with lower prevalence of FLD compared to nondrinkers and heavy drinkers. However, in our study, alcohol consumption showed no interaction with inflammatory biomarkers. Assuming the same drinking habits, subjects with FLD had higher levels of inflammatory biomarkers than those without.

## Figures and Tables

**Table 1:** Population characteristics (^§^exact Fisher's test; ^&^with 0/1-coding of variables, *P* for difference between categories).

Alcohol intake in [g/day]	0	>0–20	>20–40	>40–60	>60	*P*
*N*	100	100	100	83	49	0.0001
Men, *n* (%),	33 (33.0)	44 (44.0)	70 (70.0)	73 (88.0)	45 (91.8)	<0.0001
Total: 60,19%						
Fatty liver, *n* (%)	35 (35.0)	19 (19.0)	34 (34.0)	32 (38.6)	22 (44.9)	
Age, years	41.4 (38.9–43.9)	42.9 (40.4–45.4)	43.8 (41.2–46.3)	45.8 (43.1–48.6)	40.1 (36.5–43.6)	0.0753
BMI kg/m^2^ (SD)	26.4 (6.1)	24.82 (3.8)	25.56 (4.0)	26.34 (3.8)	26.28 (4.1)	0.0738
Actual smoker, *n* (%)	30 (30.0)	31 (31.0)	38 (38.0)	37 (44.6)	25 (51.0)	<0.0001^&^
ASA, *n* (%)	2 (2.0)	0 (0)	1 (1.0)	1 (1.2)	3 (6.1)	0.0924
Statin intake, *n* (%)	7 (7.0)	0 (0)	2 (2.0)	6 (7.2)	3 (6.1)	0.0138^§^
No sports, *n* (%)	48 (48.0)	32 (32.0)	31 (31.0)	31 (37.4)	16 (32.7)	0.084
Education < 10 years	47 (47.0)	37 (37.0)	47 (47.0)	42 (50.6)	30 (61.2)	0.0681
Hypertension, *n* (%)	16 (16.0)	12 (12.0)	11 (11.0)	24 (28.9)	9 (18.4)	0.0302^&^
Myocardial infarction, *n* (%)	2 (2.0)	0 (0)	0 (0)	1 (1.2)	1 (2.4)	0.3226^§^

**Table 2:** Arithmetic mean with standard deviation of serum concentrations of ALT, GGT, and HDL, according to alcohol consumption (*P* for difference between categories).

Alcohol intake in [g/day]	Fatty liver	0	>0–20	>20–40	>40–60	>60	*P*
HDL mmol/L (SD)	Total	1.57 (0.4)	1.70 (0.4)	1.55 (0.4)	1.61 (0.5)	1.53 (0.6)	0.0964

	Yes	1.36 (0.34)	1.43 (0.59)	1.42 (0.38)	1.43 (0.37)	1.53 (0.35)	
	No	1.67 (0.41)	1.76 (0.45)	1.61 (0.37)	1.72 (0.56)	1.52 (0.43)	

GGT U/L (SD)	Total	12.2 (7.55–16.8)	12.4 (7.79–17.1)	16.0 (11.3–20.6)	25.2 (20.1–30.3)	35.7 (29.0–42.3)	<0.0001

	Yes	18 (19.3)	19 (15.6)	16 (8.6)	28 (15.6)	57 (63.3)	
	No	9 (6.9)	11 (8.3)	16 (23.0)	23 (22.1)	18 (13.5)	

AST U/L (SD)	Total	9.53 (8.87–10.2)	9.10 (8.44–9.76)	10.1 (9.47–10.8)	11.0 (10.3–11.7)	11.2 (10.3–12.2)	0.0002

	Yes	11 (2.89)	10 (3.1)	11 (2.9)	13 (6.66)	12 (3.15)	
	No	9 (3.77)	9 (2.3)	10 (2.44)	10 (2.6)	11 (2.94)	

ALT U/L (SD)	Total	15.4 (13.9–16.9)	14.2 (12.7–15.7)	16.1 (14.6–17.6)	18.8 (17.2–20.5)	18.6 (16.5–20.7)	0.0001

	Yes	19 (7.4)	18 (6.7)	19 (7.3)	23 (9.7)	21 (8.1)	
	No	14 (8.5)	13 (6.0)	14 (6.0)	16 (6.4)	17 (5.6)	

**Table 3:** Concentrations of inflammatory markers, according to alcohol consumption (arithmetic or geometric^*^ mean with 95% confidence intervals, *P* for difference between categories).

Alcohol intake in [g/day]	Fatty liver	0	0–20	>20–40	>40–60	>60	*P*
	*N* (%) Yes/no	35/65	19/81	34/66	32/51	22/27	

CRP^*^ mg/L	Total	1.30 (1.03–1.64)	1.09 (0.87–1.38)	0.97 (0.77–1.22)	1.06 (0.82–1.37)	1.09 (0.79–1.52)	0.51

	Yes	1.55 (0.7–2.0)	2.76 (1.1–4.5)	1.50 (1.0–2.3)	1.44 (0.9–1.9)	2.25 (0.8–2.8)	0.001
	No	0.89 (0.3–1.3)	0.86 (0.1–1.1)	0.85 (0.2–1.2)	1.00 (0.3–1.3)	0.82 (0.1–1.4)	0.0002

Fibrinogen g/L	Total	3.33 (0.83)	3.07 (0.74)	3.04 (0.78)	3.07 (0.73)	3.12 (0.92)	0.072

	Yes	3.36 (3.1–3.6)	3.32 (3.1–3.5)	3.37 (3.1–3.6)	3.19 (3.0–3.4)	3.53 (3.1–3.8)	0.04
	No	3.12 (2.9–3.3)	2.96 (2.8–3.1)	2.93 (2.7–3.0)	3.11 (2.9–3.3)	3.03 (2.7–3.2)	<0.0001

oxLDL U/mL	Total	63.51 (22.0)	60.63 (20.7)	65.71 (20.4)	64.73 (20.1)	64.74 (19.4)	0.48

	Yes	67.5 (60–77)	76.2 (68–87)	76.5 (69–82)	73.3 (65–80)	72.8 (63–79)	0.16
	No	62.9 (56–68)	56.6 (52–62)	57.9 (53–62)	60.2 (55–65)	57.7 (51–65)	<0.0001

Adiponectin *µ*g/mL	Total	7.70 (4.91)	9.22 (5.46)	7.26 (3.86)	8.24 (4.65)	6.42 (3.79)	0.0046

	Yes	5.04 (4.1–5.9)	7.74 (7.0–8.6)	6.20 (5.4–7.0)	7.02 (6.0–7.7)	6.54 (5.8–7.4)	<0.0001
	No	7.59 (6.8–8.5)	8.91 (8.2–9.6)	8.48 (7.7–9.2)	9.86 (9.1–10.6)	8.07 (7.4–8.6)	<0.0001

IL-6^*^ pg/mL	Total	1.52 (1.32–1.74)	1.41 (1.22–1.61)	1.37 (1.19–1.57)	1.58 (1.36–1.84)	1.75 (1.44–2.14)	0.2405

	Yes	1.85 (1.1–2.4)	2.02 (1.4–3.2)	1.63 (1.1–2.0)	1.98 (1.0–2.8)	2.32 (1.4–3.3)	0.08
	No	1.25 (0.5–1.5)	1.35 (0.4–1.7)	1.24 (0.4–1.6)	1.33 (0.6–1.9)	1.64 (0.8–2.2)	<0.0001

E-selectin ng/mL	Total	32.7 (13.4)	32.94 (12.3)	36.39 (14.4)	37.50 (15.5)	40.62 (16.4)	0.0041

	Yes	41.3 (36–46)	40.9 (37–44)	39.8 (36–45)	40.8 (37–46)	42.3 (38–47)	0.09
	No	30.2 (27–34)	31.8 (29–35)	35.3 (31–38)	33.3 (29–37)	35.4 (29–40)	0.003

Adjusted for age, gender, BMI, smoking, physical inactivity, education, and HDL-cholesterol; ex-drinkers excluded.

## Abbreviations

ALT: Alanine aminotransferase
ASA: Acetylsalicylic acid
AST: Aspartate aminotransferase
BMI: Body mass index
CRP: C-reactive protein
CV: Cardiovascular
CVD: Cardiovascular disease
EMIL: * Echinococcus multilocularis* and Internal Diseases in Leutkirch
FLD: Fatty liver disease
GGT: Gamma-glutamyltransferase
HDL: High-density lipoprotein
IL-6: Interleukin-6
NAFLD: Nonalcoholic fatty liver disease
oxLDL: Oxidized low-density lipoprotein.
